# Mercury Levels in Hair of Domestic and Wild Animals

**DOI:** 10.3390/biology14111497

**Published:** 2025-10-27

**Authors:** Carolina Fregonesi de Souza, Robson Carlos Antunes, Vinícius José Santos Lopes, Adriana de Barros, Arlei Rodrigues Bonet de Quadros, Ricardo Lopes Tortorela de Andrade, Julio Cesar de Souza

**Affiliations:** 1 Programa de Pós Graduação em Ciências Veterinárias (PPGCVET), Universidade Federal de Uberlândia, UFU, Izaura Augusta Pereira, 286, Uberlândia 38408-192, MG, Brazil; robson.antunes@ufu.br; 2 Coordenação de Aperfeiçoamento de Pessoal de Nível Superior Brazil (CAPES), Brasília 70040-020, DF, Brazil; 3 Instituto de Ciências Agrárias e Ambientais, Universidade Federal do Mato Grosso/SINOP, Avenida Alexandre Ferronato, Sinop 78550-728, MT, Brazil; vinicius.lopes@ufmt.br; 4 Campus Aquidauana, Universidade Federal de Mato Grosso do Sul, UFMS, Rua Oscar Trindade de Barros, 740, Aquidauana 79200-000, MS, Brazil; adriana.barros@ufms.br (A.d.B.); julio.souza@ufms.br (J.C.d.S.); 5 Departamento de Zootecnia, Universidade Federal de Mato Grosso do Sul, UFMS, Av. Roraima, Santa Maria 97105-900, RS, Brazil; arlei.bonet@ufsm.br; 6 Instituto de Ciências Naturais, Humanas e Sociais, Universidade Federal do Mato Grosso/SINOP, Avenida Alexandre Ferronato, Sinop 78550-728, MT, Brazil; ricardo.andrade@ufmt.br; 7 Estudante de Medicina Veterinária—UNIDERP, Campo Grande 79003-010, MS, Brazil

**Keywords:** heavy metals, conservation, swine, sustainability

## Abstract

**Simple Summary:**

Studying the effects of mercury on swine and wild animal populations is crucial, as the latter, being free-ranging, have a greater potential for exposure to environmental contaminants such as mercury. The bioaccumulation of this metal in hair tissue indicates the degree of environmental contamination, reflecting risks to animals and the public. Assessing mercury levels in different species enables the understanding of regional and ecological patterns of exposure, which is essential for mitigation and environmental management strategies. This monitoring strengthens biodiversity conservation and food security.

**Abstract:**

This study quantified mercury (Hg) levels in the body hair of domestic and wild animals in four Brazilian states, Paraná, Mato Grosso do Sul, Goiás, and Minas Gerais, by analyzing 169 samples from sows, piglets, free-range pigs, and wild animals. The highest mean Hg concentration (274.93 ± 48.14 µg/kg) was found in wild animals in the Pantanal (MSSilvestre, Mato Grosso do Sul), followed by Minas Gerais (245.09 ± 40.27 µg/kg) and Paraná (193.0 ± 42.45 µg/kg). Levels at the GO, MGM, MSLiv, and PRV sites were significantly lower (*p* ≤ 0.05), according to the Scott–Knott test. Statistical analysis using ANOVA indicated significant variation in Hg levels between locations (F = 2.36; *p* ≤ 0.05), with homogeneity of variance (Levene’s test, *p* = 0.1772). Animals raised in confinement had lower levels than wild animals, which, due to extensive movement and contact with diverse environments, exhibited greater bioaccumulation. Lactating sows showed greater sensitivity than piglets, demonstrating an effect of animal category on metal absorption. The main sources of mercury are anthropogenic activities, such as mining and industrial processes, responsible for the environmental release of the metal. Although the detected levels do not pose an immediate risk to animal health or meat quality, they highlight the need for continuous monitoring, given mercury’s ability to bioaccumulate and affect ecosystems and food security. This work contributes to the understanding of environmental exposure to mercury in Brazil, reinforcing the urgency of effective mitigation strategies to preserve biodiversity and public health.

## 1. Introduction

Mercury (Hg) is a global environmental pollutant originating from both natural sources and human activities. Naturally, mercury occurs in deposits within the Earth’s crust, but its widespread presence in ecosystems has been significantly amplified by anthropogenic activities such as mining, industrial processes, and fossil fuel combustion, which release mercury into the atmosphere, soil, and water bodies [1,2]. Human-driven mercury emissions have significantly increased mercury levels in ecosystems, resulting in contamination of air, soil, and water bodies.

In aquatic and terrestrial food webs, mercury bioaccumulates and biomagnifies, with methylmercury (MeHg), a highly toxic organic form, posing serious risks to wildlife and human health [3,4]. Regions with intense gold mining, like parts of Brazil and the Russian Far East, face critical mercury contamination challenges, necessitating ongoing monitoring and mitigation efforts to protect biodiversity and public health [5,6].

Environmental exposure to methylmercury (MeHg), a highly toxic and bioaccumulative form of mercury, poses significant health risks to wild piscivorous fish, mammals, and birds. Controlled feeding studies have demonstrated that ingestion of MeHg at environmentally relevant concentrations results in a spectrum of toxic effects across taxa, including behavioral alterations, neurochemical disruptions, hormonal imbalances, and reproductive impairments [3].

Field investigations, particularly involving wild piscivorous bird species such as the common loon (Gavia immer), have corroborated these laboratory findings by revealing statistically significant associations between MeHg exposure and reduced reproductive success. The extent of methylmercury’s impact on wildlife populations is modulated by species-specific life history traits and regional variability in mercury deposition and methylation rates, which influence mercury concentrations in prey fish [4]. Population modeling further suggests that a reduction in mercury emissions could yield substantial recovery benefits, primarily through enhanced hatching success and survival rates of offspring, especially in vulnerable species like the common loon. While additional piscivorous species may also benefit from decreased Hg exposure, these have been less extensively studied. Overall, these findings underscore methylmercury’s pervasive ecological threat and emphasize the critical necessity for monitoring and mitigating this contaminant to safeguard wildlife health and ecosystem integrity [5].

Mercury (Hg) is a significant environmental contaminant, particularly in the studied regions of Brazil, such as the Pantanal in Mato Grosso do Sul, Minas Gerais, Paraná, and Goiás, where artisanal and industrial mining activities, in addition to deforestation for agriculture and livestock, intensify the release of Hg into the environment [6,7]. In the Pantanal, artisanal gold mining contributed approximately 2500 tons of mercury to the environment between 1980 and 1995, impacting soils, water, and local biota [7]. Agricultural and industrial expansion exacerbates the dispersion of this metal, increasing the risk of bioaccumulation in native and domestic species [6].

Pork is an essential food source for the Brazilian and global population, distinguished by its high nutritional value. It contains high-quality proteins, containing essential amino acids important for growth and health maintenance. Furthermore, it is rich in B vitamins (especially thiamine—B1), iron, selenium, zinc, and potassium, essential nutrients for the immune system, neuromuscular and cardiovascular function, and the prevention of iron deficiency anemia. Pork is a lean cut, low in calories and saturated fat, and is recommended for weight management and cardiovascular health. Regular pork consumption contributes to child development, strengthening cognitive and immune systems, and is an important source of affordable and popular protein [8].

Pigs are valuable sentinel models for monitoring environmental contaminants such as mercury due to their sensitivity and intermediate position in the food chain, feeding on feed that may contain contaminated ingredients such as fishmeal and water from local sources [9,10]. Furthermore, pigs play an essential role as food producers, being a significant source of animal protein for a large portion of the global and Brazilian populations. Thus, they provide a direct link between the bioaccumulation of contaminants in natural environments and human food safety, reinforcing the importance of monitoring them in assessing environmental and health risks. The objective of this study is to quantify mercury levels in body hair samples from different species, contributing to the understanding of mercury bioaccumulation in domestic and wild animals in Brazilian regions. Additionally, it aims to provide valuable information for developing effective mitigation and environmental management strategies.

## 2. Material and Method

**Figure 1:**
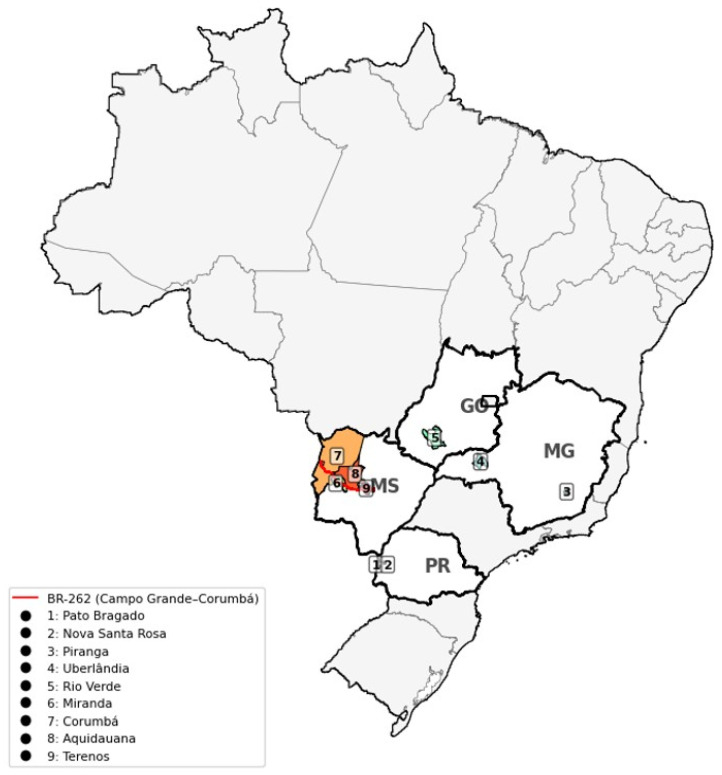
Matrices in farm maternity without the need for containment, as the females were in cages facilitating the collection process and promoting less stress to the animals.

Data collection comprised 169 analyzed samples obtained in the states of Paraná (two rural properties: PRFRI and PRV), Mato Grosso do Sul (Pantanal region and BR262 area with wildlife, MSliv site), Goiás (GO), and Minas Gerais (MGM and MGU sites). The farms located in the same state were at least 45 km apart (Figure 1).

A key aspect of this study was the choice of body hair analysis as the primary method for mercury quantification, as this technique represents a non-invasive and ethically sound approach, allowing the evaluation of live specimens without causing them harm. Considering that most of the samples were collected from live animals, the use of hair stands out as the most appropriate technique, ensuring reliable mercury quantification, preserving animal welfare, and enabling long-term ecological studies. Even animals that had recently died on the highway were haired in the same manner as live animals. All data were collected from March to June 2024, always on sunny days.

To prevent the risk of mercury contamination during the study, all samples were obtained by employees properly equipped with appropriate personal protective equipment. The rigorous use of gloves, aprons, and other specific clothing aimed to minimize direct contact with the material and avoid any cross-contamination, ensuring both the integrity of the samples and the safety of the researchers involved in the collection process. The hair sample collection was performed in a standardized manner, with all specimens collected preferably from the apex of the scapula, ensuring uniformity of the anatomical location across all samples. This uniformity was maintained even for wild animals, even those found dead after being run over, whose hair was collected from the same region, ensuring representativeness and comparability of sample data across the different categories and locations studied. These approaches reflected best practices recommended in environmental studies to ensure consistent and comparable results, essential for reliable assessments of mercury bioaccumulation in animal populations.

The inclusion of wild animals was crucial, as their widespread movement across vast areas promotes distributed sampling, enhancing the assessment of mercury bioaccumulation in natural environments. In contrast, pigs raised in semi-extensive conditions in Mato Grosso do Sul and pigs confined on farms represent controlled exposure conditions, enabling the comparison of mercury accumulation in different ecological contexts. Thus, the aim was to determine whether free-range animals have higher mercury concentrations than domesticated animals in regulated environments, considering their unrestricted access to diverse areas, including potentially contaminated ones. Despite the taxonomic diversity of the specimens, differences between species were not a limiting factor, since the focus was on mercury dose, sensitivity, and absorption, regardless of species-specific variations.

The results corroborate the hypothesis that environmental exposure plays a critical role in mercury bioaccumulation, highlighting the need for continuous monitoring to assess contamination risks and implement effective mitigation strategies to ensure ecosystem integrity and food security. The sample groups were divided into confined and free-range animals. In the first group, samples were collected from lactating sows in the farrowing phase and from piglets in the weaning phase. In the second group, samples were collected from domestic pigs raised in extensive systems, which were rounded up at night for protection from predators and released during the day near the cities of Aquidauana and Miranda. Wild animal samples were obtained from the carcasses of animals run over on the BR262 highway (Aquidauana and Corumbá region).

Wildlife collections took place along BR262—which crosses the Pantanal Biome—and included representative species of local reptiles, birds, and mammals. Notable reptiles include the jararaca (*Bothrops moojeni*), vine snake (*Phrelodryas matogrossensis*), green snake (*Erythrolamprus typhlus*), red boa (*Epicrates cenchria*), and anaconda (*Eunectes notaeus*). Also collected were tijucos (*Tupinambis palustris*), scavengers and birds of prey such as the vulture (*Coragyps atratus*) and the western guan (*Bubo virginianus*), as well as mammals typical of the Pantanal, including: tapir (*Tapirus terrestris*), capybara (*Hydrochoerus hydrochaeris*), white-lipped peccary (*Pecari tajacu*), wild pig (*Tayassu pecari*), hoary fox (*Cerdocyon thous*), raccoon (*Procyon cancrivorus*), coati (*Nasua nasua*), giant anteater (*Myrmecophaga tridactyla*), lesser anteater (*Tamandua tetradactyla*) and six-banded armadillo (*Euphractus sexcinctus*).

On farms where animals were kept in intensive housing, samples were collected from sows of varying ages, eliminating the need for deliberate restraint, as the sows were housed in dedicated stalls to prevent accidents with the piglets, facilitating collection and minimizing stress. Biological material—body hair—was obtained using sterile surgical scissors and surgical gloves, removed from the dorsal region, a procedure also replicated for the piglets.

Each sample was individually packaged in a sterile container, properly labeled “P” for sows, “L” for piglets, and “S” for wild animals, accompanied by their scientific and common names. To ensure the removal of external contaminants that could compromise the measurement of mercury intrinsic to the hair, the samples were subjected to three individual washes using a sieve, distilled water, and mild detergent. The chosen washing protocol proved adequate for removing particles and surface residues, avoiding interference with the analytical results. After drying the samples in an oven at 50 °C, they were placed in properly labeled Petri dishes for weighing. This was performed on a Shimadzu analytical balance with a sensitivity of 0.1 mg.

The glassware used (test tubes, volumetric flasks, funnels) was previously decontaminated by simple washing with tap water, followed by triple rinsing, immersion in a 5% potassium permanganate solution for 30 min, rinsing again, and immersion in 20% hydrochloric acid for another 30 min. This was completed with a rinse test in distilled water, ensuring complete elimination of contaminants. For chemical analysis, the weighed samples were placed in digestion tubes containing 1 mL of distilled water, 5 mL of concentrated sulfuric acid, and 2 mL of perchloric nitric acid. The tubes were heated in a 40-unit digestion block at 230 °C for 30 min, promoting the decomposition of organic matter and the release of mercury for analysis. After cooling to room temperature, the contents were diluted in a 25 mL volumetric flask with distilled water, leaving it ready for reading.

The samples were separated individually in sterile containers, identified as P for sows, L for piglets, and S for wild animals (with scientific name). Then, they were washed individually with distilled water and neutral detergent and dried. After drying, the samples were weighed on an analytical balance (Shimadzu) with a precision of 0.1 mg, placed in digestion tubes, and submerged in a solution composed of 1 mL of distilled water, 5 mL of sulfuric acid, and 2 mL of nitric acid and perchloric acid. The test tubes were heated in a digestion block with a capacity of 40 units at 230 °C for 30 min [11]. After heating, the samples were left to return to room temperature and then diluted in 25 mL volumetric flasks with distilled water [2,12,13]. The determination of total mercury (THg) in the resulting digestion solutions was performed by atomic absorption spectroscopy with cold vapor generation, using the Agilent AA240FS Varian Inc. (Palo Alto, CA, USA/Mulgrave, Victoria, Australia) equipment, coupled to the VGA77 accessory. The stock solution used for curve calibration was traceable to NIST, provided by Specsol^®^ Estonia, Tallinn, Estonia.

The detection limit (LD) and quantification limit (LQ) of the method were determined from blank samples, where LD was calculated as the mean of ten blanks plus three times the standard deviation, and LQ was calculated as the mean of ten blanks plus ten times the standard deviation. Validation showed that the mean of the blanks in the solution was 0.011 µgL^−1^, with an LD of 0.0451 µgL^−1^ and an LQ of 0.124 µgL^−1^, corresponding to 5.64 µgkg^−1^ and 15.48 µgkg^−1^ in dry samples, respectively.

To assess the accuracy and precision of the analysis, we analyzed the Certified Reference Sample of human hair ERM-DB001 (European Reference Material) Northeastern Belgium, sample 588, produced by the Institute of Reference Materials and Measurements of the European Commission’s Joint Research Centre, which was certified with 365 ± 28 µg kg^−1^ Hg. In our laboratory, the average value recovered was 388 µg kg^−1^ (106% of the certified value), with a coefficient of variation of 3.43%, proving the quality of the chemical analysis by the methodology used.

Statistical Analysis:

The statistical analysis was performed using the R program [14], version 4.3.3. Normality conditions were verified using the Kolmogorov–Smirnov test. The Levene test was used to verify if the samples had equal variance. Therefore, when not met, the data were transformed log, as presented later. For group comparison, one-way ANOVA was performed with parametric tests and analysis of variance. The statistical model used was:

The model used in this study was as follows:

*Y_ijk_* = µ + *L_i_* + *C_j_* + *ϵ_ijk_* (1)

where *Y_ijk_* is the estimated amount of Hg µ is the general mean *L_i_* is the fixed effect of the *i*th location (*i* = 1…7) *C_j_* is the fixed effect of the *j*th category (Sow; Piglet; Wild animal) *ϵ_ijk_* is the random error with mean 0 and variance *σ*^2^. The limit of *p*-value considered in this study to reject the null hypothesis was *p* ≤ 0.05.

## 3. Results and Discussion

A total of 169 samples, including those from wild animals and farmed pigs, were collected from regions such as Goiás, Minas Gerais, Paraná, and Mato Grosso do Sul. The analysis used atomic absorption spectrophotometry to measure mercury (Hg) concentrations, highlighting significant disparities between groups. Notably, wild animals from Mato Grosso do Sul and pigs from specific farms in Minas Gerais had the highest mercury levels. These results are in line with studies by [15,16], which indicate that mercury exposure is not limited to areas with a history of mining; For example, a study in indigenous villages in the Brazilian Amazon revealed that 42% of the fish consumed had total mercury levels above the safe limits established by the World Health Organization. This highlights a broader environmental problem, where both aquatic and terrestrial species are at risk from mercury pollution, which can have serious implications for human health and biodiversity. The presence of mercury in animal hair serves as an important biomarker for assessing environmental contamination and highlights the urgent need for monitoring and management strategies to mitigate these risks in various ecosystems.

The results reveal a significant disparity in mercury concentrations between farmed pigs and wild animals in the Pantanal. While farmed pigs showed high mercury levels, indicating possible contamination associated with controlled feeding and the rearing environment, wild animals showed variable concentrations, reflecting the complexity of natural ecosystems and the different sources of mercury exposure. This disparity highlights the importance of monitoring and controlling the presence of mercury in both domestic and wild environments, aiming to mitigate negative impacts on public health and biodiversity conservation of the region. The results of the analysis of mercury (Hg) concentration in different locations revealed distinct distribution patterns, as evidenced by the means and standard errors presented in Table 1.

This variation in mercury concentration between locations can be attributed to a series of anthropogenic, environmental, and geographic factors that influence the biogeochemical cycle of mercury and its availability in the different environments studied. The primary sources of mercury include human activities such as mining, coal burning, and other industrial processes. These activities release mercury into the environment, which can be converted into methylmercury, a highly bioavailable form.

To assess mercury levels in wildlife, we can use biomaterials from animals, such as body hair, which serve as indicators of exposure to the metal. This approach allows us to quantify mercury bioaccumulation in different species. This study utilizes both domestic and wild species to evaluate mercury bioaccumulation. The selection of these species provides a broader understanding of exposure and bioaccumulation patterns in different environments. Research on mercury bioaccumulation in animals is current and relevant, especially in regions with economic activities that potentially release mercury into the environment. Previous studies in Brazil and elsewhere highlight the importance of monitoring and mitigating mercury exposure.

Mercury (Hg) levels observed at the different sampled sites in Table 1 and ranged from 88.8 ± 32.3 µg kg^−1^ in GO to 274.9 ± 24.8 µg kg^−1^ in wild samples from MS, with the highest values recorded in MG.

According to the toxicological parameters established by the FAO/WHO, the Provisional Tolerable Weekly Intake (PTWI) for methylmercury was set at 1.6 µg/kg bw/week. Subsequent reviews by the European Food Safety Authority (EFSA) adjusted this value to a Tolerable Weekly Intake (TWI) of 1.3 µg/kg bw [10,17]. Furthermore, the Codex Alimentarius establishes a maximum limit for total mercury in fish of 0.5 mg/kg (500 µg/kg), while the United States, through the FDA/EPA, adopts an action level of 1 mg/kg (1000 µg/kg) for methylmercury in aquatic products [18].

Other countries, such as Japan and Australia, maintain more restrictive limits for predatory species, reflecting greater caution regarding trophic bioaccumulation [19].

Comparing these references with the average values obtained in this study, it is observed that all samples presented Hg concentrations below the maximum permitted limits for foods intended for human consumption (≤274.9 µg kg^−1^ compared to limits of 500–1000 µg kg^−1^). However, the interpretation that such levels would be “non-harmful” should be considered with caution.

International limits are based on edible tissues (primarily fish) and do not necessarily apply directly to terrestrial mammal tissues, such as hair or internal organs. Furthermore, Hg toxicity strongly depends on the predominant chemical form—methylmercury being the most bioaccumulative and neurotoxic species [13]. Furthermore, even levels below dietary limits can result in sublethal or chronic ecological effects, particularly in environments where there is continuous trophic biomagnification.

Therefore, although the observed concentrations are below regulatory food safety limits, the detection of average levels above 200 µg kg^−1^ in certain areas (MG_U, PR_FRI, MS_Silvestre) reinforces the need for continuous environmental monitoring and additional studies on the chemical form of Hg and its ecotoxicological effects. These findings corroborate the literature that points to the persistence and environmental mobility of mercury as risk factors for biota, even at apparently moderate concentrations. Similar studies have been conducted in Brazil and other countries, emphasizing the need for continued research on mercury bioaccumulation.

The results of the ANOVA analysis performed to investigate the differences in mercury concentrations between the sampled locations indicated statistically significant variation between the groups (F = 2.36, *p* ≤ 0.05). This confirms the hypothesis that mercury concentrations vary significantly between different locations of the data sampling. This result is consistent with previous studies that reported the influence of geographical, environmental, and anthropogenic factors on the distribution of mercury in different regions [12].

The Levene test was performed to verify the homogeneity of variances between groups, revealing a *p*-value of 0.1772. This non-significant result indicates that the assumption of equal variances was satisfied, strengthening the reliability of the ANOVA results [13]. The analysis of variance revealed differences between locations. Normal data by the Levene test, where *p* = 0.1772. Those that presented significant differences (ANOVA F = 2.36, *p* ≤ 0.05), rejecting H0 and accepting H1, that there is mercury presence in the collected samples.

The quantification of Hg in the body hair of different species in this study is within the range of values found in other mammals, despite the species investigated in the literature being from different taxonomic groups.

Although this study did not directly measure mercury levels in soil, water, or plants in the regions studied, it should be noted that mercury contamination in Brazil, including the states of Goiás, Minas Gerais, Paraná, and Mato Grosso do Sul, is associated with anthropogenic sources, such as mining (legal and illegal), agricultural, and industrial activities that release the metal into the environment. In the Pantanal (Mato Grosso do Sul), for example, artisanal gold mining is a major source of mercury, which is dissipated in the soil and water, subsequently being incorporated into the local food chain. Similarly, in the states of Goiás, Minas Gerais, and Paraná, where pig farming is an important activity (with Goiás and Minas Gerais experiencing intense production expansion and Paraná leading in pig production), the environmental presence of mercury can originate from both industrial processes and agricultural use, influencing animal exposure. Thus, the mercury detected in the hair of these pigs reflects environmental exposure from these multiple contaminant sources, reinforcing the need for continuous monitoring and evaluation to protect animal and public health [13].

**Figure 2:**
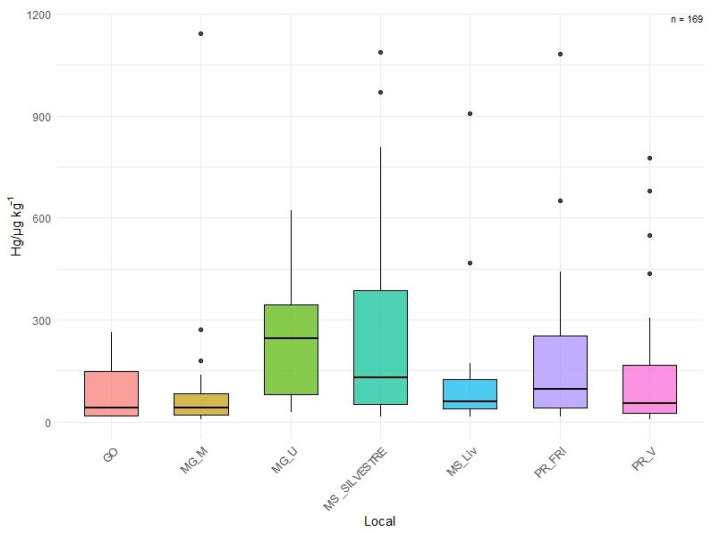
Results of the mercury concentration analysis (Hg) in different locations (N = 169) by Location (GO: Goiás, MGM: Minas Gerais, MGU: Minas Gerais, MSSilvestre: Mato Grosso do Sul, wild fauna, MSliv: Mato Grosso do Sul, free-range pigs, PRFRI: Paraná, PRV).

The results show a significant disparity in mercury concentration between farm-raised pigs and wild animals. The MGM location presented a point quite outside the cloud of the others (outlier), Figure 2. Perhaps a more sensitive animal. While farm-raised pigs presented high levels of mercury, indicating possible contamination associated with controlled feeding and breeding environments, wild animals demonstrated variable and higher concentrations. These individuals, by moving freely and having access to diverse locations, can access locations with higher incidences of this metal. This response may reflect the complexity of natural ecosystems and different sources of mercury exposure. This disparity highlights the importance of monitoring and controlling mercury presence in both domestic and wild environments, aiming to mitigate negative impacts on public health and biodiversity conservation.

The results of the mercury concentration analysis (Hg) in different locations revealed distinct distribution patterns, as evidenced by the means and standard error of the mean presented in Figure 2.

When examining the mercury concentration averages for each location, it was observed that individuals from the MSSilvestre location presented the highest average (274.93 ± 48.14 µg/kg), followed by MGU and PRFRI, with averages of 245.09 ± 40.27 µg/kg and 193.0 ± 42.45 µg/kg, respectively. Despite significant variation, there were significant differences between some. This variation in mercury concentration between locations can be attributed to a series of factors such as anthropogenic and environmental changes or access to regions with higher concentrations of mercury (geographic), which can influence the biogeochemical cycle of mercury and its availability in different environments, promoting greater absorption by living organisms that come into contact with this metal.

These results suggest that these areas may be subject to significant sources of mercury contamination, possibly due to the influence of industrial activities, urbanization, and environmental degradation. On the other hand, the locations GO, MGM, MSLiv, and PRV exhibited lower mercury concentration means, all accompanied by the same letter bin the Scott-Knott test.

**Figure 3:**
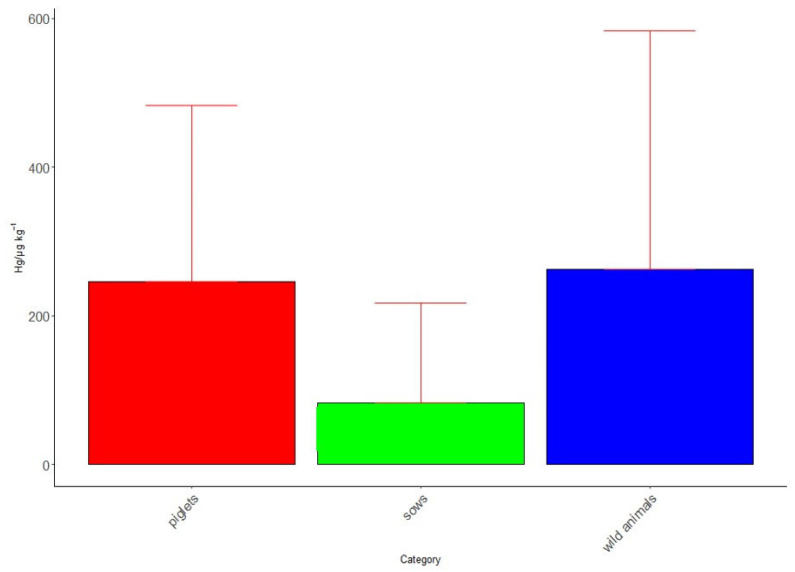
Exposure levels of categories piglets, sows and wild animals to mercury presence.

When comparing the sensitivity of mercury by category, it was observed that wild animals and sows revealed a higher incidence than piglets. The sensitivity to Hg can be observed in Figure 3.

The values obtained from these assessments involving domestic pigs and wild animals suggest a lesser influence of anthropogenic sources of mercury in these areas, which can be attributed to their geographic location, land use patterns and lower industrial activity. These results corroborate those presented by [20,21].

A study involving the concentration of mercury in the body hair of 13 ocelots in the interior and surroundings of the Serra da Canastra National Park (PNSC) considered that the animals were exposed to contamination by mercury, even though they were distant from important sources of this element and that small mammals are also exposed. The assimilation of mercury by the ocelots occurred due to their feeding on small mammals and within the PNSC, the total mercury concentrations were higher than in the surrounding regions, possibly due to the local life, food, and/or water [22].

The results revealed highly relevant information, demonstrating that even when levels do not reach extreme values, there is cause for concern. This finding highlights the pressing need for rigorous monitoring of mercury levels in facilities producing pork for human consumption. These findings align with reports by [1], who describes the existence of two distinct forms of mercury poisoning: acute and chronic.

The chronic exposure in species of fish and wild animals, related to the bioaccumulation of mercury in the environment, continues to be a problem. In domestic animal species, the clinical signs of involvement of the nervous, gastrointestinal, respiratory, and reproductive systems are typical and influenced by the form, dose, and duration of exposure. The diagnosis can be confirmed based on the clinical picture, histopathological findings, and results of tissue analysis for mercury concentration [2,12,23].

Due to the permanent damage to tissues and the significant implications for food safety, treatment options may be limited and often discouraged. Despite the samples of wild animals being from carcasses found on the roadside, the most likely cause of death for these was likely to be roadkill and not mercury poisoning. In production pigs, none presented a possible mercury poisoning. However, monitoring these species is essential for the overall control of mercury incidence in different species.

Jenkins et al. [24] identified a mean of 1.1 µg/g of body hair in squirrels from urban areas, while in rural areas with less direct human impact, the mean was 0.43 µg/g of body hair. Ref. [25] evaluated normal mercury concentrations in the body hair of wild animals, such as raccoons, otters, and American mink in non-industrialized areas, varying between 1 and 5 µg/g of body hair.

Other studies also contribute to understanding the variation in mercury concentrations in wild fauna. Ref. [26] reported a mean of 0.18 ug per gram of body hair in black bears in Idaho, USA, suggesting a natural origin for this contamination. Yet Jenkins et al. [24] found mercury concentrations in the body hair of gray squirrels varying from 0.07 to 9.2 µg/g, while in the body hair of sled dogs, the values varied from 0.14 to 15.8 µg/g, which is still within the normal range for this species.

In Brazil, studies also revealed a wide variation in mercury concentrations in ocelots, sampled in the Serra da Canastra National Park MG, presenting concentrations of mercury between 0.62 and 9.8 µg/g of body hair. Studies on contamination in the body hair of jaguars found concentrations varying from 673 to 917 µg/g in areas influenced by mining in Mato Grosso, while in Mato Grosso do Sul, where there is no known influence of mining, the concentrations varied from 29.7 to 23.3 µg/g [23,27].

The samples of wild fauna analyzed in Mato Grosso do Sul presented a mean concentration of 274.9 ± 323.4 µg/g, although they were not of the same species, indicating possible contamination that requires a more detailed study of the origin. Ref. [21] observed concentrations ranging from 0.27 to 4.80 µg/g in tamanduas (Myrmecophaga tridactyla) on highways in Mato Grosso do Sul, which were below the range associated with adverse effects in other species.

These results highlight the complexity of mercury contamination in wild fauna and the need for continuous monitoring to evaluate the risks to human and animal health. The variation in mercury concentrations reflects the diversity of environmental and anthropogenic factors that can influence the exposure and accumulation of this heavy metal in different regions and species [21,22].

It is crucial to note that the results obtained may have significant implications for human health and the ecosystem, since mercury is a known environmental contaminant with adverse effects on health, particularly when bioaccumulated in the food chain. In addition, analyzing the presence of mercury in swine can serve as an environmental indicator, helping to monitor environmental contamination by this metal. Mercury contamination can occur through various sources, including industrial activities, mining, and natural processes. Therefore, diagnosing the presence of mercury in the body hair of porcine is essential to ensure food safety, protect public health, and monitor environmental contamination, thus contributing to sustainable practices and preserving animal and human health.

However, a more in-depth study is necessary to identify the specific sources of mercury contamination in each location, as well as to evaluate the potential impacts on public health and environmental conservation. Continuous monitoring strategies and mercury contamination mitigation measures must be implemented to protect human health and preserve the integrity of affected ecosystems. These results have significant implications for environmental management and public health, highlighting the need to continuously monitor mercury concentrations in different geographic areas and implement mitigation measures to reduce human exposure and protect the health of affected ecosystems. These results corroborate those reported [28,29].

Assessment of mercury levels in various wild species is crucial not only to understand the extent of environmental contamination but also to evaluate potential risks to biodiversity and ecosystem health. Wild animals often occupy higher trophic levels, making them susceptible to mercury bioaccumulation and biomagnification, which can lead to toxic effects that affect reproduction, neurological function, and survival. Monitoring mercury in wildlife also serves as an early warning system for ecosystem disturbances, especially in biodiverse and environmentally sensitive regions such as the Pantanal. In addition, there is growing concern about mercury contamination in food production systems, particularly in domestic animal species such as swine.

Mercury exposure in swine, primarily through contaminated food, water, or soil, poses direct risks to food safety and public health, since pork represents a significant source of animal protein for human consumption. Therefore, continuous monitoring of mercury levels in production animals is essential to ensure the safety of the food supply chain and to implement effective environmental and agricultural management practices to reduce mercury ingress into the human diet.

## 4. Conclusions

Diagnosing the presence of mercury in pigs in Brazil is extremely important given the potential impacts on public health and food safety. The detection of this metal in their hair indicates the animals’ exposure to environmental contaminants, such as polluted soil and food, directly impacting meat quality and, consequently, posing a risk to consumers’ food safety. Although the levels detected in the regions studied are not currently considered harmful, further investigation is urgently needed to quantify and monitor mercury deposition in these animals, aiming to protect human and animal health. The identification of mercury in pigs is strategically important because it demonstrates regional and interspecies variations, highlighting the influence of environmental and anthropogenic factors on contamination. Wild animals and piglets presented higher mercury concentrations compared to sows, revealing the role of habitat and diet in the bioaccumulation of the metal. Although the observed levels do not pose an immediate risk, they reinforce the importance of continuous monitoring and the implementation of mitigation strategies to reduce mercury exposure, protecting biodiversity and food quality, and promoting long-term environmental sustainability and public health.

## Figures and Tables

**Table 1:** Comparison of means using the Scott-Knott test.

Local	Hg Concentration (µg kg^−1^)
GO	88.8 ± 32.3 b
MG_M	96.7 ± 15.8 b
MG_U	245.1 ± 13.6 a
MS_Liv	127.1 ± 15.3 b
MS_Silvestre	274.9 ± 24.8 a
PR_FRI	193.0 ± 18.7 a
PR_V	157.7 ± 16.0 b

Different letters in the same collum have a minimum *p* < 0.05.

## Data Availability

The data cannot be shared because it is part of a research program and is still being used. Perhaps this could happen in the distant future.
